# Adenosine—A drug with myriad utility in the diagnosis and treatment of arrhythmias

**DOI:** 10.1002/joa3.12453

**Published:** 2020-12-18

**Authors:** Anunay Gupta, Yash Lokhandwala, Nitish Rai, Amit Malviya

**Affiliations:** ^1^ Department of Cardiology Vardhman Mahavir Medical College and Safdarjung Hospital Delhi India; ^2^ Department of Cardiology Lokmanya Tilak Municipal General Hospital Mumbai India; ^3^ Department of Cardiology North Eastern Indira Gandhi Regional Institute of Health and Medical Sciences Shillong India

**Keywords:** anti‐arrhythmic drugs, atrioventricular block, supraventricular tachycardia, ventricular tachycardia

## Abstract

Adenosine has been used in the emergency treatment of arrhythmia for more than nine decades. However, cardiologists are often unfamiliar about its basic mechanism and various diagnostic and therapeutic uses, considering it mainly as a therapeutic drug for supraventricular tachycardia. This article discusses the role of adenosine relevant to emergency physicians, cardiologists, and electrophysiologists. Understanding of the mechanisms of adenosine and its electrophysiological effects is discussed first, followed by dosing, side effects, diagnostic, and therapeutic uses. Finally, the role of adenosine in the electrophysiology laboratory is discussed.

## Graphical abstract

Adenosine is a multifaceted drug with myriad utility in the diagnosis and treatment of arrhythmias.

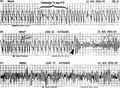

## 1 INTRODUCTION

A fundamental in cardiac electrophysiology is to understand the mechanism of arrhythmia. Adenosine, a naturally occurring endogenous metabolite, formed by the degradation of adenosine triphosphate (ATP), not only terminates but also helps in defining the mechanism of several arrhythmias, which was discovered in 1929.^1^, ^2^ ATP gets metabolized into adenosine after intravenous administration and was used in the treatment of cardiac arrhythmias.^3^ Both have similar efficacy and clinical effects; however, adenosine is more stable at room temperature^4^ and is, therefore, preferred over ATP. It terminates AV nodal dependent arrhythmias like atrioventricular nodal reentrant tachycardia (AVNRT) and atrioventricular reentrant tachycardia (AVRT).^1^ It also terminates many focal atrial tachycardias and ventricular arrhythmias due to triggered activity. Since it induces conduction block in the AV node, adenosine has diagnostic utility in patients with narrow QRS complex tachycardia.^5^ Similarly, adenosine could be of the most value in hemodynamically stable wide QRS tachycardia (WQRST) with 1:1 VA conduction, when there is difficulty to differentiate supraventricular tachycardia with aberrancy from ventricular tachycardia (VT). In the electrophysiology laboratory, adenosine plays a critical role in unmasking the accessory pathway conduction,^6^ both before and after ablation and it also has a role in pulmonary vein isolation.^7^ Recently, an entity named *neurohumoral syncope* has been proposed to differentiate from classical vasovagal syncope because of the presence of low serum adenosine in the former.^8^

In this review, we aim to provide an insight into the electrophysiological effect of adenosine, and in‐depth analysis of the utility of adenosine in the field of cardiac electrophysiology in diverse clinical scenarios.

## 2 ELECTROPHYSIOLOGICAL EFFECTS AND MECHANISM OF ACTION

**FIGURE 1:**
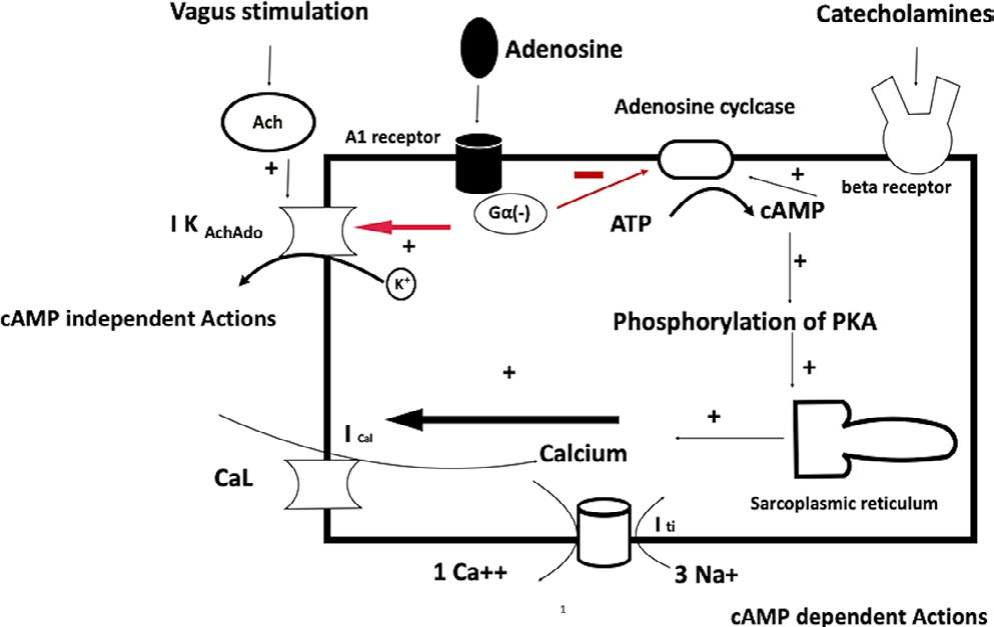
Effect of adenosine on cardiac cells through A1 receptors can be either cAMP‐dependent or independent. Activation of potassium channels (negative chronotropy and dromotropy) is cAMP‐independent while inhibition of hyperpolarization‐activated (funny I_f_) current (phase 4 depolarization) and I_CaL_ are cAMP‐dependent. The figure is adapted from Lerman et al^12^

There are predominantly two types of G‐protein‐coupled adenosine receptors (GPCRs) in the heart: A1 on cardiomyocytes, responsible for its electrophysiological effects, and A2 on endothelial and vascular smooth muscle cells, which mediate coronary vasodilation.^9^ A1 receptors which are coupled with Adenosine depresses the sinoatrial (SA) node activity, AV nodal conduction and atrial contractility^10^ Adenosine mediated cardiac actions (Figure 1) are i) cAMP‐independent (direct), due to the activation of G_i/o_ which leads to release of G_βγ_ (beta‐gamma) subunits; hence, activating G protein‐coupled inwardly rectifying adenosine sensitive potassium channels (which are also stimulated by acetylcholine), also known as IK _AchAdo_ and ii) cAMP‐dependent (indirect or antiadrenergic effects), which lead to the inhibition of inward calcium current (I_CaL_) and transient inward sodium current (I_ti_).^11^, ^12^ G_α_ (alpha) subunit of the A1 receptor leads to the inhibition of cAMP production, which is responsible for the inhibition of sympathetic response through beta receptors and inward calcium current.^11^, ^12^

### 2.1 Sinoatrial node

**FIGURE 2:**
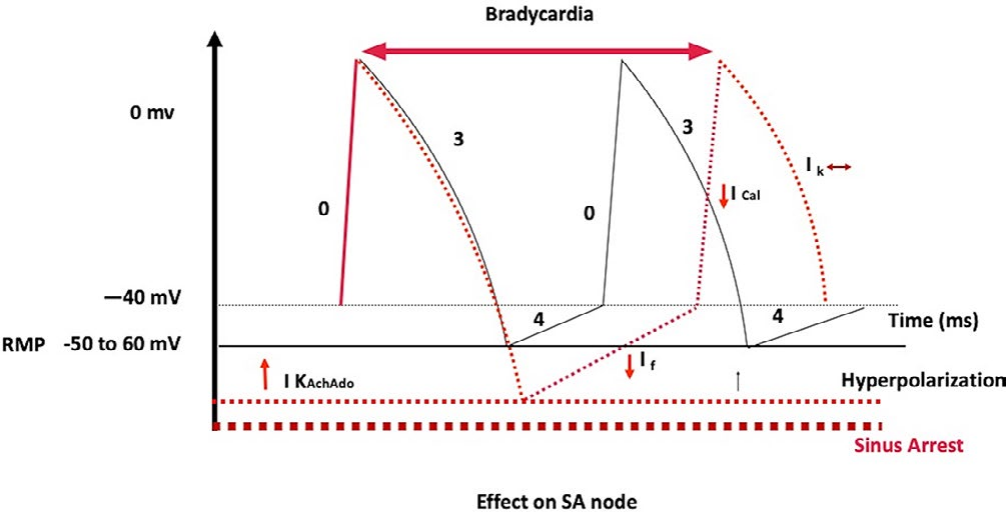
Effect of adenosine on SA node in red. Adenosine leads to hyperpolarization (dotted line ‐ red color) of the resting membrane potential; therefore, decreases phase 4 slope of diastolic depolarization. Excessive hyperpolarization can lead to sinus arrest (Dotted line ‐ brown color). It also inhibits hyperpolarization‐activated (funny I_f_) current (phase 4 depolarization) and I_CaL_ (Phase 1) as an indirect effect through cAMP inhibition in the presence of sympathetic stimulation

Stimulation of I K_AchAdo_ (direct effect) leads to an increase in K efflux; hence, there is hyperpolarization of SA nodal cells and a decrease in the sinus rate *(negative chronotropy)*.^10^, ^11^ Excessive hyperpolarization can also lead to sinus arrest. Furthermore, it inhibits hyperpolarization‐activated (funny I_f_) current (phase 4 depolarization) and I_CaL_ (Phase 1) as an indirect effect through cAMP inhibition in presence of adrenergic stimulation (Figure 2). Adenosine results in a biphasic effect on heart rate, an initial period of sinus bradycardia is followed within seconds by sinus tachycardia. The latter response is due to adenosine‐mediated activation of carotid body chemoreceptors (presence of adenosine receptors) resulting in respiratory stimulation and activation of pulmonary stretch receptors.^13^ Thus, this increase in heart rate is not seen in patients with autonomic failure.

### 2.2 Atrium

**FIGURE 3:**
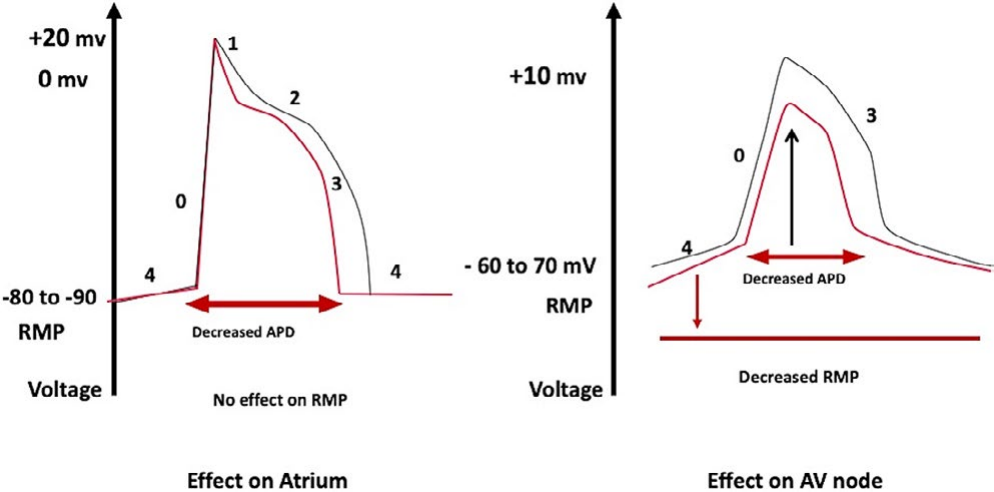
Effect of adenosine on the atrium on the left and atrioventricular (AV) node on the right. Adenosine decreases action potential duration (APD) hence refractory period of atrium without any effect on resting membrane potential (RMP) while decreasing RMP (more negative) of AV node without any effect on APD. [Red is the effect of adenosine and black is control]. The figure is adapted from the Lerman et al^15^

Adenosine has a *negative inotropic* effect on atrium. The atrium differs from the SA node and AV node because there is no phase 4 spontaneous depolarization and the resting membrane potential remains steady throughout the diastole without any role or presence of I K_AchAdo_. Therefore, activation of I K_AchAdo_ leads to the shortening of action potential duration and decreased refractoriness of atrial muscle during phases 2 and 3, (Figure 3) which can lead to the precipitation of atrial flutter and fibrillation.^14^

### 2.3 AV node

There is hyperpolarization of the membrane potential due to the activation of I K_AchAdo_. Initially, this leads to depression of the upstroke of the action potential in AV nodal (N) cells and complete abolition later, which leads to the decreased conduction in the AV node (*negative dromotropy*), resulting in PR prolongation and AV block (Figure 3).^15^, ^16^

### 2.4 His‐Purkinje system (HPS)

Adenosine has a minimal direct effect on the His‐Purkinje system under resting conditions. However, adenosine inhibits increased automaticity of HPS under conditions of increased catecholamines, suggesting an indirect effect.^17^

### 2.5 Ventricles

Human ventricular myocytes do not have adenosine‐sensitive potassium channels; hence, adenosine has no effect on the ventricular action potential. Nonetheless, it antagonizes the actions of catecholamine on L‐type calcium current (I_CaL_) and on the transient inward current (I_TI_) (Figure 2), which decreases the amplitude of delayed after‐depolarizations (DADs) and suppresses triggered activity induced by agents known to increase the concentration of cellular cAMP.^18^ Therefore, adenosine can terminate idiopathic right and left ventricular outflow tract VT dependent on cAMP‐mediated triggered activity.^12^, ^18^

### 2.6 Accessory pathways (APs)

Adenosine usually does not affect conduction through typical APs. However, adenosine can block conduction in the APs, which have long conduction time (decrementally conducting) such as atriofascicular pathways and permanent junctional reciprocating tachycardia (PJRT).^19^ Atriofascicular pathways contain AV nodal tissue, they are also expected to slow down with verapamil.^20^ However, in a detailed case study, there was no acute response to verapamil.^21^ The pathways in PJRT can respond to both adenosine and verapamil; pathways that respond only to adenosine‐induced hyperpolarizing K+ current likely comprises of depressed fast‐Na+ channel tissue, while those who respond to both adenosine and verapamil are AV node like structures.^22^ Adenosine is known to decrease antegrade effective refractory period(ERP) of bypass tracts,^23^ which can induce VF in susceptible patients with short APERP.^24^ Another explanation that has been postulated is the reflex sympathetic activation due to adenosine‐induced peripheral vasodilation leading to a fall in blood pressure.^13^

Fasciculoventricular pathways are rare variants of accessory pathways arising from his bundle or bundle branch and inserting into ventricle.^25^ These accessory pathways do not cause any clinical arrhythmia; therefore, do not need ablation.^26^ Nodoventricular/nodofascicular pathways are rare; they bypass a portion of the AV node and insert directly into the crest of ventricular septum and conduction system, respectively. Because the AV node has decremental conduction, these pathways are also sensitive to adenosine.^25^

## 3 DOSING OF ADENOSINE AND SIDE EFFECTS

Adenosine is available as a sterile saline formulation for intravenous (IV) administration. Adenosine should be stored below 25°C but *should not be refrigerated*. It is delivered as a rapid IV bolus followed immediately by saline flush. Electrophysiological effects may not manifest if administered slowly as the half‐life of adenosine in the blood pool is less than 10 seconds due to rapid metabolism by endothelial cells and red blood cells. For this reason, utmost care should be taken, and blood should be absent in the syringe. Effects of adenosine are manifested within 10 to 20 seconds and 30 seconds via central and peripheral routes, respectively.^5^

The initial minimum recommended dose in adults is 6 mg IV bolus. Subsequent doses should be increased by 6 mg to a maximum of 18 to 24 mg, the incremental doses if needed, are to be given after 1 to 2 minutes each time. Adenosine triphosphate (ATP) is used in Japan instead of adenosine and is equally efficacious. Dosing is the rapid intravenous infusion of 5 to 10 mg of ATP, and if ineffective, increase the dose to 20 mg. The administration should be in a central vein if possible, eg, the elbow is preferable to the hand. In children the recommended initial dose is 0.1 mg/kg, with 0.1 mg/kg increments if necessary, up to a maximum of 0.5 mg/kg. Continuous ECG recording (preferably 12 lead) or storage on a central monitor is essential to define the mechanism of arrhythmia; hence, it should be a part of the protocol while administering adenosine. Adenosine can be safely used during pregnancy; the initial bolus is 6 mg, followed by 2 boluses of 12 mg if necessary and a maximum bolus dose of 24 mg.^27^

Dipyridamole inhibits the uptake of adenosine, potentiating its effects. Therefore, the dose of adenosine should be reduced in patients on dipyridamole.^28^ Similarly, post‐cardiac transplant patients require a reduction in the dose of adenosine due to denervation‐induced supersensitivity to the drug.^29^ ^.^ Methylxanthines and xanthine derivatives (theophylline) have a dose‐dependent blockade of adenosine receptors and hence, a higher dose of adenosine is required in these patients; but adenosine should be avoided in patients with a history of bronchospasm.

The half‐life of adenosine is very short, so the desired as well as the unwanted effects are generally short lasting usually less than 60 seconds. Adverse effects are varied and common with adenosine, which reflects the ubiquitous expression of adenosine receptors in the human body and circulatory system. Side effects are more common with IV infusion rather than boluses; however, rarely these require any intervention. The most common side effects are facial flushing, dyspnea, and chest pressure. (Table 1) Ventricular premature complexes and run of NSVT are relatively common but without any adverse consequences after tachycardia termination, which is considered due to increased sympathetic discharge.^30^, ^31^ There are a few case reports of life‐threatening adenosine‐induced VT/VF due to the bradycardia following adenosine; all of these patients had acquired or congenital long QT syndrome.^32^, ^33^ When used in WPW syndrome, especially for preexcited AF, adenosine can lead to VF because of a combination of i) AV nodal block with more conduction down the accessory pathway, ii) Reduction of accessory pathway refractory period, and iii) Increased ventricular irritability due to reflex sympathetic stimulation. *Therefore, a defibrillator should always be available bedside while administering adenosine*.

**TABLE 1:** Side effects of adenosine

Common (>10%)	Uncommon (1%–10%)	Rare (<1%)
Flushing Dyspnea Chest pressure Bradycardia PVCs/ NSVT	Light‐headedness Dizziness Tingling in arms Nausea Numbness	Bronchospasm Hypotension Blurred vision Sweating Asystole Atrial fibrillation Ventricular fibrillation Hypersensitivity Seizure

## 4 DIAGNOSTIC USE OF ADENOSINE

The property of adenosine to depress and block the AV nodal conduction makes it a useful diagnostic tool in a variety of clinical scenarios.

### 4.1 Presence of Latent pre‐excitation

**FIGURE 4:**
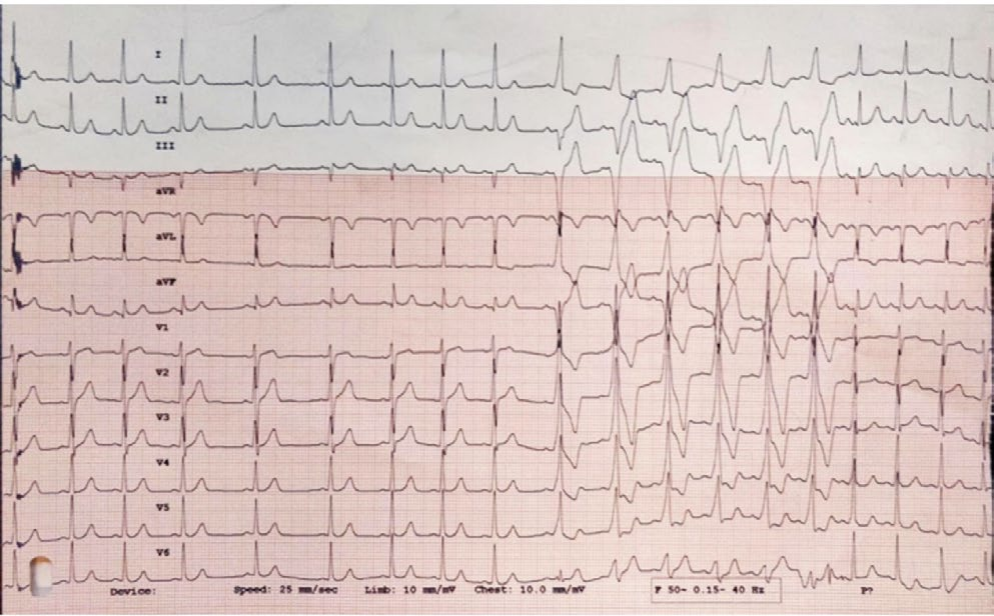
Preexcitation being unmasked by adenosine in a young male with a history of palpitations without documented tachycardia. The delta waves are negative in leads II/III and positive in V1; this was a left posterior accessory pathway, later ablated successfully

In patients with suspicion of an accessory pathway, it is often difficult to decipher the delta waves (pre‐excitation) due to the relative competing conduction velocities in AV nodal (faster) and accessory pathway (slowly conducting) or long distance of the accessory pathway (left lateral pathway) from the SA node.^34^ Adenosine, by prolonging AV nodal conduction, allows more conduction through the accessory pathway (adenosine usually has no effect on accessory pathway except for atriofascicular pathways or PJRT) (Figure 4).

### 4.2 Fasciculoventricular pathway

Fasciculoventricular pathways are different from typical APs because these patients are not at the risk of sudden cardiac death or tachycardia episodes.^25^ Administration of adenosine injection can help in making the differential diagnosis. In response to adenosine stress, there will not be a greater degree of pre‐excitation; following adenosine‐induced AV block, junctional beats with the same degree of pre‐excitation as in sinus rhythm can be seen.^35^ Suzuki T et al used adenosine test to identify fasciculoventricular pathways, if AV block occurred without a change in QRS waveform or if the PR interval was prolonged by more than 40 msec without a change in QRS morphology.^36^ Fasciculoventricular pathway in association with PRKAG2 mutation usually shows the lack of response to adenosine and develops sinus bradycardia without AV block. These patients are at increased risk of AV block and atrial arrhythmias on follow‐up compared to those without PRKAG2 mutation.^26^

### 4.3 Unmasking dual AV nodal physiology

**FIGURE 5:**
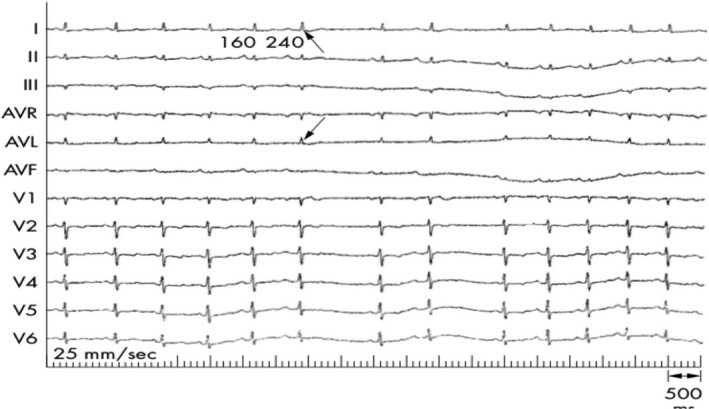
Atrioventricular nodal echo (retrograde P wave at the end of the QRS complex) during the adenosine test, shown by an arrow, which is preceded by a PR jump (denoted by numbers in milliseconds)^37^

The presence of dual AV nodal physiology can be exposed in sinus rhythm by the administration of adenosine (Figure 5).^37^ Furthermore, adenosine can even rarely induce AV nodal reentrant tachycardia.^38^ Adenosine has differential effects on antegrade fast and slow AV nodal pathways, with early and sustained suppression of antegrade fast pathway conduction.^39^ Dual AV node physiology manifests as a sudden increase in the PR interval, resulting from the block in fast and shift to slow pathway. A single dose (12 mg) of intravenous adenosine administered during sinus rhythm in patients with documented narrow QRS tachycardia without manifest preexcitation can identify dual AV node physiology in the surface ECG recording at the bedside with a high negative predictive value. A positive adenosine test is identified by either PR jump (more than 50 msec) or AV nodal echo (retrograde P wave at the end of the QRS complex).^37^

### 4.4 Narrow complex tachycardia

**FIGURE 6:**
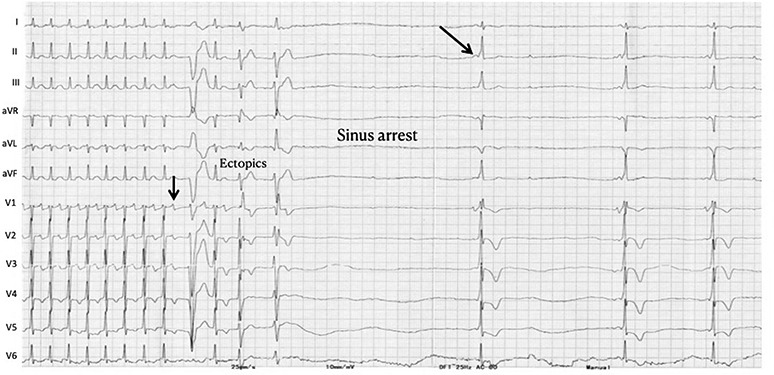
Narrow QRS tachycardia with intermediate RP interval terminates with a P wave after adenosine, suggesting AVRT. Ectopics followed by sinus arrest are seen after termination, both of which are common after adenosine. Later, preexcitation is seen suggestive of a left free wall pathway which was inapparent during tachycardia

**FIGURE 7:**
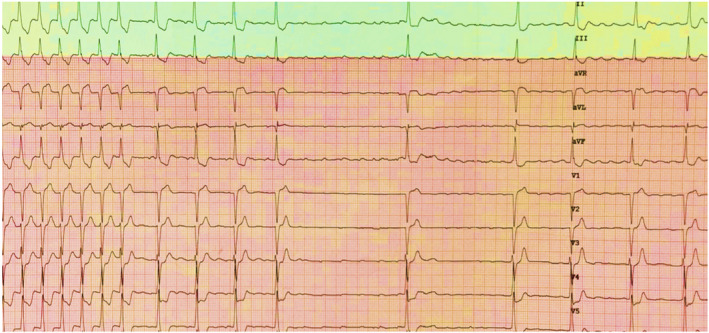
Atrial tachycardia/atrial flutter being unmasked by adenosine. The initial part shows 1:1 AV conduction, followed by a complete AV block. Later, the arrhythmia degenerates into atrial fibrillation (better seen in lead aVF)

**FIGURE 8:**
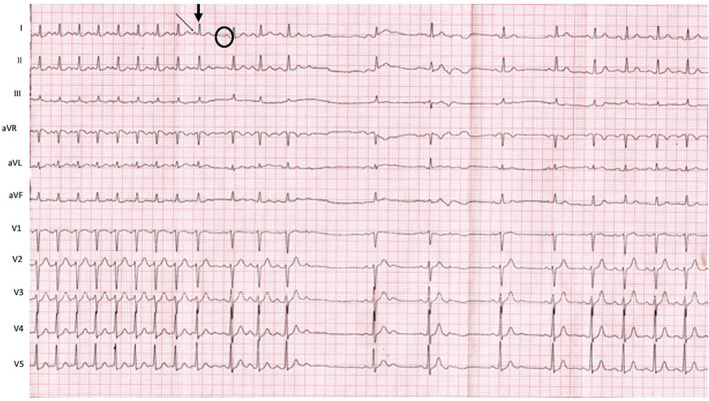
On the left side of the tracing there is narrow complex tachycardia with long RP interval with P waves (slanting arrow) which are positive in inferior leads ruling out AVRT and AVNRT. Also, narrower as compared to sinus beats (circle). Tachycardia terminates after a QRS complex (arrow) suggestive of adenosine sensitive atrial tachycardia

**FIGURE 9:**
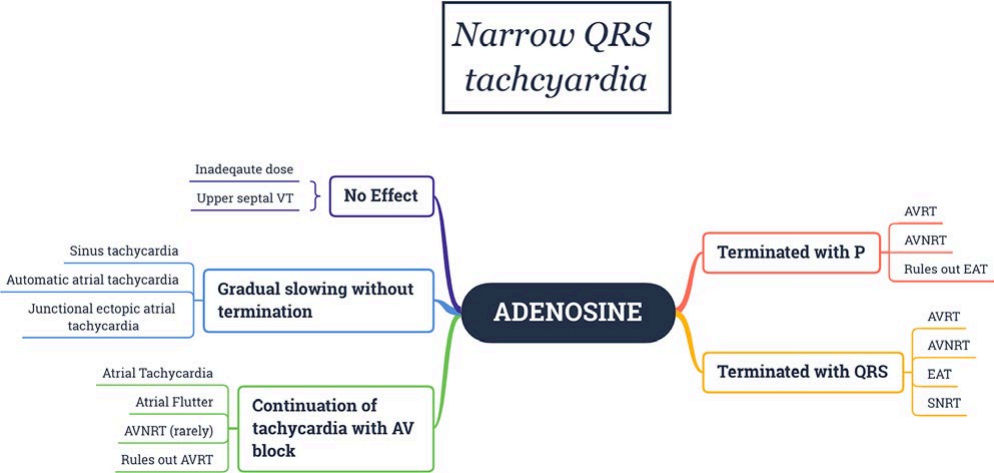
Role of adenosine in the differential diagnosis of Narrow QRS tachycardia^44^

The differential diagnoses of narrow complex tachycardia are AVNRT (typical or atypical), AVRT, and atrial tachycardia (AT) and rarely junctional ectopic tachycardia (JET). Adenosine creates a conduction block in the antegrade slow pathway in AVNRT and in the AV node in AVRT due to rapidly conducting accessory pathway; therefore, both tachycardias usually terminate with a P wave. However, atypical AVNRT (fast‐slow variant) can terminate due to the block in the retrograde direction in the slow pathway, hence, with a QRS complex. Also, adenosine can unmask latent pre‐excitation (Figure 6) after termination, if the accessory pathway can conduct antegradely. If adenosine administration leads to AV block without the termination of tachycardia, AVRT/AVNRT can be ruled out. AV nodal block might unmask underlying atrial tachycardia (Figure 7) or even can terminate adenosine sensitive AT.^39^ Termination of AT usually occurs prior to the onset of AV block; therefore, P wave will be followed by a QRS complex (Figure 8). If SVT terminates repeatedly after a P wave, it rules out AT. Triggered activity‐mediated ATs terminate due to anti‐adrenergic effects of adenosine,^40^ while micro‐reentrant ATs terminate because the site of origin (periannular/perinodal) resemble AV nodal tissue electrophysiologically, which might explain adenosine responsiveness.^41^ Hence, it is wrong to consider AT termination with adenosine as a specific sign of a triggered activity mechanism.^41^, ^42^, ^43^ Figure 9 summarizes the role of adenosine in the differential diagnosis of narrow complex tachycardia.^44^

Occasionally, orthodromic AVRT can have a decrementally conducting accessory pathway instead of a rapidly conducting accessory pathway, which is sensitive to adenosine and leads to a permanent form of junctional reciprocating tachycardia. Therefore, during adenosine administration tachycardia can terminate with either P wave (block in the slow pathway) or QRS complex (block in the accessory pathway).

Junctional ectopic tachycardia (JET) is a rare arrhythmia in adults, which occurs most commonly after cardiac surgery and presents usually as narrow complex tachycardia.^45^ However, it is more often seen in infants and children where it occurs spontaneously. The exact mechanism of JET is still an enigma but, appears to be non‐reentrant.^46^ JET due to triggered activity is likely to respond to adenosine, while those due to abnormal automaticity are adenosine insensitive.^47^

### 4.5 Wide QRS tachycardia

**FIGURE 10:**
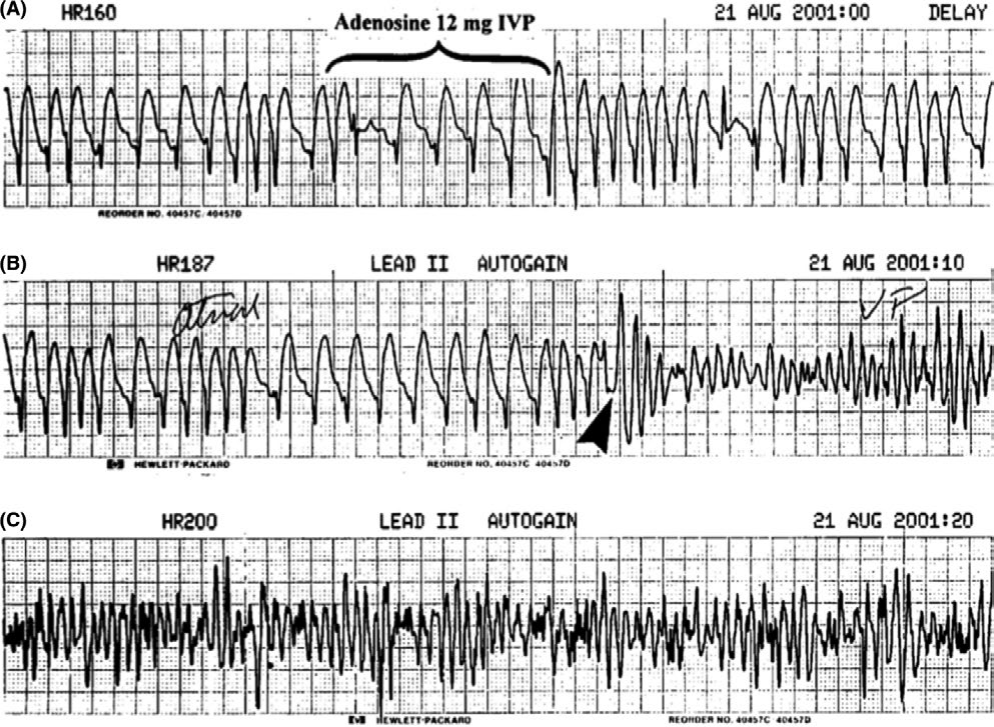
Electrocardiogram monitor strips. Panel A shows pre excited atrial fibrillation. Adenosine (12 mg) denoted by an arrow was administered in the emergency department. Panel B shows the development of ventricular fibrillation after adenosine administration^24^

**FIGURE 11:**
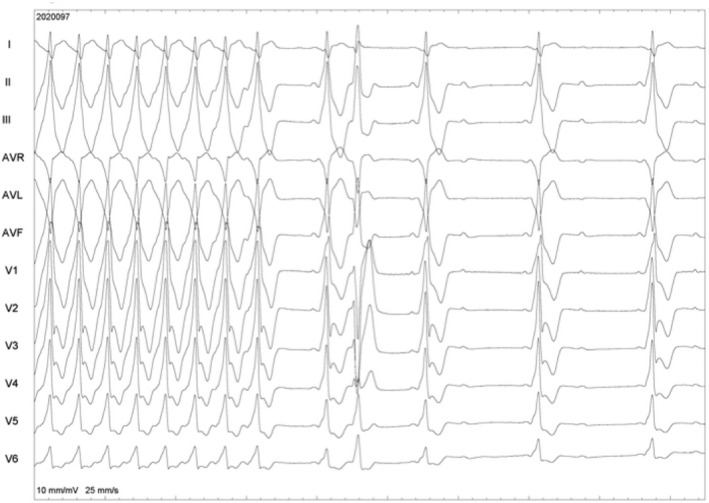
ECG in the initial part shows WQRST with RBBB morphology in V1 and an inferior axis. Differential diagnoses are a pre excited tachycardia and ventricular tachycardia. Adenosine leads to the termination of tachycardia, following which preexcitation with an identical QRS is seen, followed by AV block. This was an antidromic tachycardia due to the adenosine‐sensitive accessory pathway

WQRST is often a diagnostic dilemma on the surface ECG. The differential diagnosis of WQRST is VT, SVT with aberrancy, SVT with underlying bundle branch block, and antidromic AVRT. Adenosine, with its property of blocking the AV nodal conduction, can be utilized in such cases. If a WQRST is supraventricular in origin, adenosine should terminate the arrhythmia if the AV node is involved or produce AV block to reveal the underlying atrial rhythm like in SVT/Atrial flutter with aberrancy. Conversely, during VT with 1:1 retrograde conduction, adenosine can bring out ventriculoatrial block or dissociation. Importantly, as explained before, adenosine can terminate idiopathic RVOT VT;^12^, ^18^ therefore, termination of WQRST is not diagnostic of SVT. Because adenosine can trigger atrial fibrillation, it can have adverse hemodynamic consequences in antidromic AVRT. Similarly, it should be avoided as a diagnostic agent for irregular WQRST because it may cause VF during pre‐excited atrial fibrillation (Figure 10).^24^ Antidromic AVRT from typical APs get terminated from adenosine due to block in AV node which is retrograde limb. Nonetheless, typical APs can demonstrate adenosine sensitivity, although it is rare (Figure 11).

### 4.6 Neurohumoral syncope (Low adenosine syncope)

A low‐adenosine phenotype of neurohumoral syncope has recently been identified and can be diagnosed by low serum adenosine levels.^8^ These patients suffer syncope without prodromal symptoms with a normal baseline ECG and do not have any structural heart disease. The typical mechanism of syncope is an idiopathic paroxysmal atrioventricular block or sinus bradycardia, most often followed by sinus arrest. These patients are highly susceptible to endogenous adenosine; chronic treatment of these patients with theophylline, a non‐selective adenosine receptor antagonist can prevent syncopal recurrences. In a small study of 16 patients with low‐adenosine syncope, chronic theophylline therapy was highly effective in decreasing syncope recurrence who had an idiopathic AV block.^48^ Whether long‐term theophylline therapy can completely avoid pacemaker implantation is currently unknown.

## 5 THERAPEUTIC USES OF ADENOSINE

### 5.1 Sinus node reentrant tachycardia

Adenosine by virtue of hyperpolarizing the membrane reduces the pacemaker current (I_f_), can terminate sinus node reentrant tachycardia. It terminates with a QRS complex and adenosine‐induced AV block occurs *after* tachycardia termination.

### 5.2 Focal atrial tachycardia

There is still a general misconception among cardiologists that adenosine has little effect on focal atrial arrhythmias. However, adenosine not only plays a therapeutic role by terminating focal atrial tachycardia but also helps in defining the mechanism and location of atrial tachycardia. Termination of atrial tachycardia by adenosine has been considered as a criterion to classify that tachycardia as due to triggered activity^40^ but reentrant perinodal ATs are also adenosine sensitive, especially those that are ablated from the non‐coronary sinus.^41^, ^49^ It has been suggested, with embryologic dissections, that the remnants of the retro‐aortic node are responsible, and this may also explain sensitivity to adenosine.^50^ The effect of adenosine is more pronounced in the AT circuit than in the AV node and tachycardia terminates before an effect on the AV node is visible.^41^ Similarly, ATs arising in the mitral and tricuspid annuli are also adenosine sensitive, likely related to the presence of remnant AV node like tissue.^40^, ^51^, ^52^ Criteria used for diagnosing triggered activity were inducibility and termination with programmed stimulation, and a discrete electrogram (<20% of tachycardia cycle length) recorded from the site of earliest activation; but these are not specific for triggered activity.^52^ Therefore, termination of AT by adenosine has been wrongly considered to be characteristic (100% sensitive and specific) for triggered activity.^52^

### 5.3 AVRT/AVNRT

Adenosine is almost always effective in terminating supraventricular tachycardia in which the AV node is part of the circuit. Adenosine creates a conduction block in the slow pathway in AVNRT and in the AV node in AVRT. Adenosine also terminates APs with decremental conduction properties such as atriofascicular tachycardia and PJRT.^19^

### 5.4 Ventricular tachycardia (VT)

Majority of VTs are insensitive to adenosine. Adenosine does not affect VT due to micro‐reentry, macro‐reentry, and enhanced automaticity as discussed above.^10^ An exception to this, in which adenosine is highly effective are VTs which are due to cAMP mediated triggered activity due to after depolarizations. These type of tachycardias originates from the outflow tracts and are induced by exercise or sympathetic surge. Adenosine terminates these tachycardias (Figure 1) due to its antiadrenergic effects (cAMP dependent).^18^

## 6 ROLE OF ADENOSINE IN ELECTROPHYSIOLOGY LAB

Adenosine plays an indispensable role in the electrophysiology lab because of its electrophysiological properties of the activation of IK _AchAdo_ and blocking conduction in the AV node.

### 6.1 Unmasking conduction over concealed APS

Even though para‐Hisian pacing maneuvers or differential RV pacing can help us in differentiating conduction over AV node or a septal accessory pathway, often this is not clear due to rapid AV nodal conduction or fusion between AV nodal and accessory pathway conduction.^53^, ^54^ In such instances, adenosine can unmask conduction because it will not have any effect on a typical accessory pathway. In a recently published study, the persistence of ventriculoatrial conduction during ventricular pacing prior to ablation in response to adenosine (≤24 mg) was found to be 90% sensitive and specific for detecting a retrograde AP and inducible AVRT; however, although uncommon, decremental APs are also adenosine sensitive and can lead to fallacious response.^55^ Other limitation is the inability to achieve VA block in 9% of patient in this study due to insensitivity of retrograde AV nodal pathway to adenosine.^55^ Adenosine is also helpful when mechanical trauma leads to the disappearance of accessory pathway conduction during mapping prior to ablation.

### 6.2 Confirmation after successful ablation of APS

As discussed above, adenosine inhibits AV nodal conduction (both anterograde and retrograde) without any effect on typical APs; hence, adenosine induced AV and VA block after ablation is considered a rapid and reliable method to assess successful ablation and should be a routine protocol in the EP lab^56^ (Online supplement Figure S1).

Similarly, adenosine can unmask dormant conduction after successful ablation and identify APs, which are at increased risk of recurrence.^6^ The mechanism of adenosine‐induced AP conduction is hypothesized to be AP excitability recovery due to membrane hyperpolarization. The presence of dormant AP conduction is a significant predictor of recurrence requiring repeat ablation.^6^ Additional ablation to eliminate dormant AP conduction can lead to decreased recurrence rates. Adenosine can unmask incomplete ablation and can help in diagnosing the development of decremental conduction properties in typical APs during catheter ablation. Hluchy Jet al^57^ reported two patients with manifest pre‐excitation due to typical APs, who developed markedly slow adenosine sensitive decremental properties after radiofrequency ablation due to injury‐induced marked anisotropy. This was eliminated by repeat ablation.

### 6.3 Pulmonary vein isolation

It is essential to ensure effective and durable pulmonary vein isolation, as recurrences rate is high, particularly in persistent AF which are often related to the recovery of pulmonary vein to left atrial conduction.^7^ Adenosine has been used to identify the presence of dormant conduction (DC) after pulmonary vein isolation. Activation of IK _AchAdo_ leads to membrane hyperpolarization and restoration of the excitability threshold. If cells are partially damaged after ablation, adenosine will help in the recovery. Two prospective randomized control studies, the ADVICE trial (Adenosine Following Pulmonary Vein Isolation to Target Dormant Conduction Elimination) and the UNDER‐ATP trial (Unmasking Dormant Electrical Reconduction by Adenosine Triphosphate) have assessed the role of adenosine after AF ablation.^58^, ^59^ In the ADVICE trial, dormant conduction was associated with increased risk of atrial tachyarrhythmia recurrence. Furthermore, elimination of dormant conduction by additional ablation reduced recurrent atrial tachyarrhythmias by >50%. There was a waiting period of 20 minutes after pulmonary vein isolation. In patients with spontaneous vein reconnection, adenosine was administered after eliminating reconnection. Similarly, the UNDER‐ATP trial assessed the role of adenosine; however, it did not show any benefit in the primary endpoint of recurrent atrial tachyarrhythmia at 1 year of follow‐up. There were differences in the methodology of both trials, which was responsible for different results. First, the ADVICE trial had a fixed waiting time of 20 minutes after the completion of PVI and the dose of adenosine was variable and depend on the ability to produce AV block or sinus pause; in the UNDER‐ATP trial, the dose of adenosine was the same for every patient, regardless of the effect produced and the waiting time was variable (median was 43 minutes). Second, in the UNDER‐ATP trial, one‐third of the patients had persistent or long‐standing AF, while the ADVICE trial had patients of paroxysmal AF only. Therefore, it appears that dose titrated adenosine to achieve AV block or 3‐second pause after 20 minutes of PVI can improve outcomes of AF ablation. Nonetheless, the use of adenosine increases the procedure duration and costs. The administration of adenosine 20 minutes following PV isolation is a Class IIb recommendation in the 2017 AF ablation Guidelines. ^60^

### 6.4 Atrial flutter ablation

Even after achieving a successful complete bidirectional isthmus block, the recurrence rate of flutter was reported to be high (9.3%) in a recent meta‐ analysis.^61^ Adenosine provoked the resumption of conduction across the cavotricuspid isthmus in 9% of the patients and identified patients at increased risk of recurrence.^62^ The mechanism of the activation of conduction is the same as discussed above in atrial fibrillation. It was observed that a negative adenosine challenge immediately after successful isthmus ablation with bidirectional block, or after the abolition of dormant conduction with further ablation, strongly predicted the absence of subsequent spontaneous reconnection within 30 minutes; therefore, this avoids waiting for 30 minutes postablation to look for recovery of conduction.^63^

## 7 CONCLUSION

To summarize, adenosine, a multifaceted drug available in the armamentarium of a cardiologist for more than nine decades has many uses. Having an in‐depth knowledge of the mechanism of action and its electrophysiological properties is a prerequisite in exploiting its wide diagnostic and therapeutic role in cardiac arrhythmias.

## CONFLICT OF INTEREST

None.

## Supporting information

Supplementary Material

## Data Availability

The data underlying this article are available in the article and in its online supplementary material.
