# Supplementary figures and images for: Adenosine—A drug with myriad utility in the diagnosis and treatment of arrhythmias
Source: J Arrhythm. 2020 Dec 18;37(1):103–12. doi: 10.1002/joa3.12453 (PMC7896475; doi:10.1002/joa3.12453)

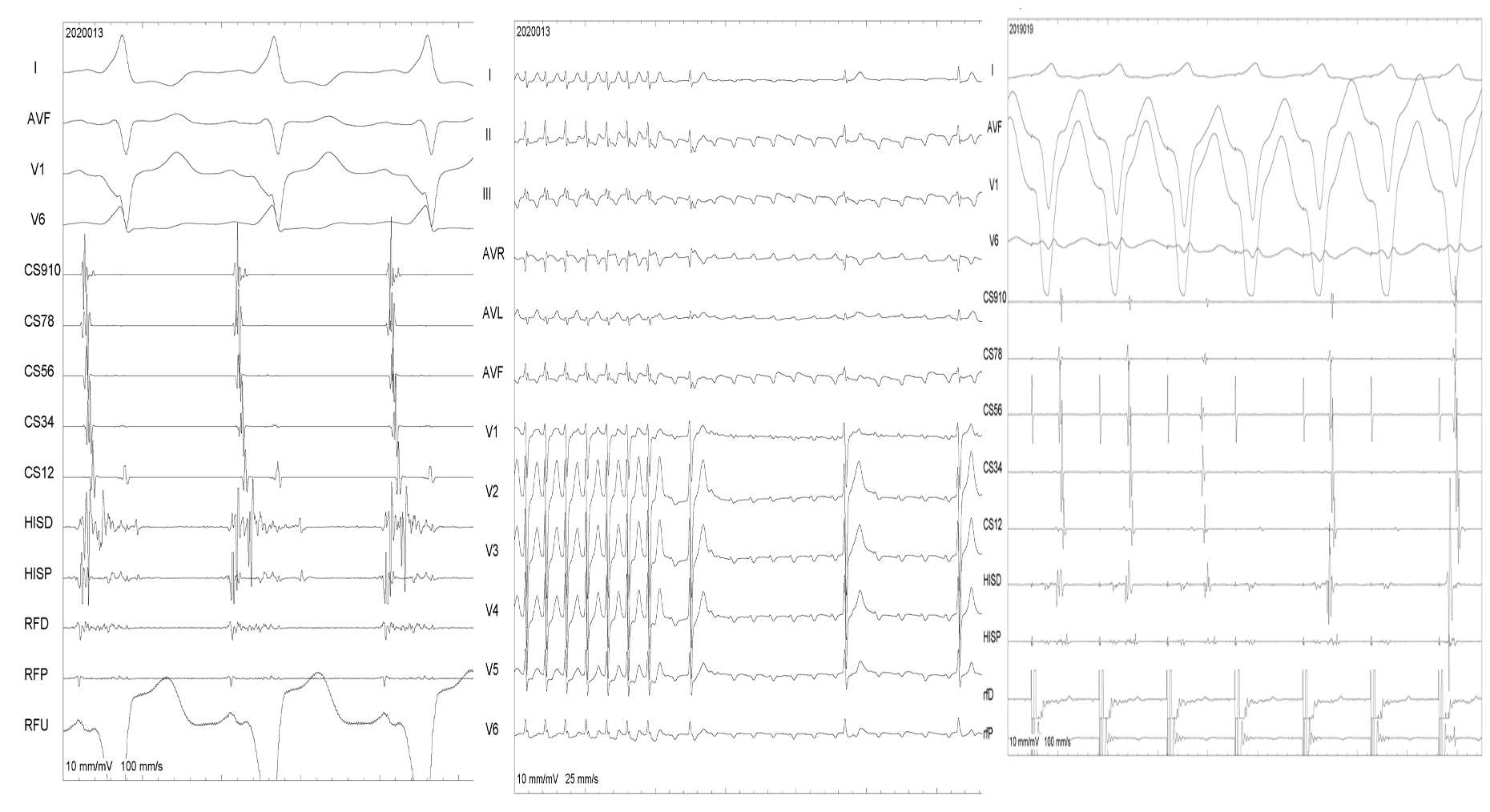

Supplement: Supplementary file 1 — Supplementary Material [file JOA3-37-103-s001.png]
